# Barriers to care: Caregivers’ accounts of raising a child with a communication disorder

**DOI:** 10.4102/sajcd.v71i1.1036

**Published:** 2024-08-12

**Authors:** Nicole Cooke, Clare Harvey

**Affiliations:** 1 Department of Psychology, Faculty of Humanities, University of the Witwatersrand, Johannesburg, South Africa

**Keywords:** childhood disability, communication disorder, barriers to care, lived experiences, mother, parental caregiver, Johannesburg, South Africa

## Abstract

**Background:**

There exists a dearth of research on the psychological experiences of childhood communication disorders. Caregivers of these children are one source who can provide us with this information since the child exists within a system. Literature on the experiences of caregivers of children with communication disorders, specifically in the South African city context, is lacking.

**Objectives:**

The article presents the reported experiences of six parental caregivers raising a child with a communication disorder within Johannesburg.

**Method:**

Data were collected via semi-structured interviews and underwent an interpretative phenomenological analysis.

**Results:**

Five themes are presented: feeling out of control; barriers to accessing services; caregivers left speechless; a misunderstood disability; and relinquishing control: *‘I needed to be a mommy’*.

**Conclusion:**

The caregivers relayed an initially negative experience in raising a child with a communication disorder, marred with worry and fear for the future. In accessing services, they narrated their experiences of multiple barriers to access, including an overreliance on the private sector, financial barriers, time constraints, limited resources and poor information on available resources. Eventually, the caregivers were able to identify strengths, skills and capabilities within their children and themselves that allowed for adjustment and coping.

**Contribution:**

The findings highlight the importance of considering how barriers to care may marginalise and seclude parental caregivers. Over time, the caregivers were able to empower themselves through building their own knowledge and reformulating their narratives by challenging assumptions and retitling the source of disability as a product of the failure of society.

## Introduction

Communication disorders form a broad category of deficits in language, speech or communication (American Psychological Association [APA], 2022). The APA considers a communication disorder a developmental disability (2022). ‘Disability’ is an enduring physical and/or mental impairment that impacts the daily life functioning of an individual, which can include communication (APA, 2022). Similarly, ‘disorder’ is described as distress that interferes with functioning (APA, 2022); therefore, ‘disorder’ and ‘disability’ are used interchangeably in this article.

A South African study documented that 13% of children between the ages of 6 months and 12 months were diagnosed with complications in communication (Van der Linde et al., 2016). It is estimated by the World Health Organization (WHO 2023) that 16% of the world’s population have a disability. According to StatsSA in 2011, the prevalence rate of disability in South Africa was 7.5% and may be even higher now. Although disability is a common feature of the world’s population, a large proportion of people living with disabilities stem from low-income countries, including South Africa (Goodley, 2017). Children are more vulnerable to disability, exposed to environmental hazards and increasingly diagnosed with disorders; consequently, the number of children with disabilities is increasing globally (Albrecht et al., 2001; Anderson et al., 2007). Although disability is widespread, the field of disability studies is a relatively young academic discipline (Vergunst, 2016). Children with communication disorders may struggle to express themselves and notably lack a voice in the already existing dearth of psychological research on this topic (Cunningham et al., 2017; Simms & Jin, 2015).

The article presents the lived experiences of parental caregivers who are raising children with a communication disorder within the South African city context. For many families in South Africa, childcare arrangements occur in contexts of low rates of marriage and cohabitation; therefore, family systems are varied, fluid and flexible (Mkhwanazi et al., 2018). The term ‘caregiver’ is preferred to accommodate these contextually-driven variations. ‘People/children with disabilities’ and ‘disabled people or children’ are used interchangeably here. Person-first language, although commendable, has been unsuccessful in its efforts to combat stigma. Therefore, ‘disabled people/children’ is acceptably used as a descriptor and term of social experience, status and identity, as well as self-declaration and collective declaration (APA, 2022).

Exploring the context within which disability is experienced offers insight into the interrelationship between disability and the broader systemic components. According to the WHO (2023), there continues to exist ongoing prejudice and stigmatisation of disability, with disability being associated with exclusion and disadvantage. In South Africa, disability predominantly continues to be situated in the individualistic medical model – disability is viewed as an issue pertaining to the person in which the disability is solely caused by physical injury or damage to the body (Beighton & Wills, 2019) – with a focus on welfare that stigmatises and prejudices against people with disabilities and their families (Watermeyer, 2019). Despite progress being made in terms of legislation and policy reform in South Africa, children with disabilities and their families often experience multiple deprivations, a lack of basic needs and invisibility in the policy agenda (ACPF, 2011). Children with disabilities and their families came under the greatest influence of the medical model. Thus, medical, educational and social service professionals exercised oppressive power over disabled people, while disabled people bore the responsibility for their care (Longmore, 2016). The overreliance on professionals’ authority often overrules the self-knowledge, empowerment and independence of disabled children and their caregivers, shrouding them with uncertainty (Case, 2000; Longmore, 2016).

Scholars are encouraged to move away from the medicalised model of disability research and produce research that offers a more accurate understanding of the lived experiences of disabled people (Rembis, 2019). Additionally, exploring the context within which disability is experienced offers insight into the interrelationship between disability and access to services (Law et al., 2019). Caregivers are believed to demonstrate issues pertinent to children with disabilities and their voices are considered relevant in research. Exploring caregivers’ experiences of raising a child with a disability is an important aspect of disability studies, providing insight into the daily-lived experiences and perceptions of caregivers to understand the impact of raising a child with disabilities. Research in the field of caregiving in South Africa rarely focusses on resources and support systems available to caregivers, with caregivers reportedly experiencing significant financial, mental and occupational stress (Stjernswärd & Hansson, 2020). Furthermore, in underresourced countries such as South Africa, caregivers may face a lack of service provision and necessary information in order to understand and best deal with their child’s communication disability (Mkabile & Swartz, 2020). The prejudices against children with disabilities and their caregivers are reflected in the dearth of research related to the experiences of family systems of children with communication disorders in the South African context (Grinker et al., 2012; Simms & Jin, 2015). In this context, literature on caregivers of children with communication disabilities was found to be largely inadequate and lacking in empirical validity (Mkabile & Swartz, 2020).

Furthermore, Saville Young and Berry (2016) found that the daily-lived experience of caregivers is often ignored and replaced with a focus on their practical needs such as healthcare requirements and policy implementation. In cases where communication disabilities within the context of South Africa have been addressed, the reported experiences of caregivers were omitted and attention was paid to school intervention and readiness (Maluleke et al., 2019). The overreliance on the medical model is reiterated in the literature where there exists a dearth in inquiry related to the experiences of family systems of children with communication disorders in the South African context (Grinker et al., 2012). Furthermore, research conducted on disability is often geared towards the experiences of the disabled individual and not necessarily the caregivers’ experience of taking care of the disabled individual (Maphosa & Chiwanza, 2021). However, Khoza-Shangase (2019) conducted a useful study on the reported experiences of caregivers on service delivery for their children with hearing impairments. In addition, Kara and Harvey (2016) explored mothers’ experiences of raising their deaf children. However, the focus in the current article is on caregivers of children with communication disorders that are predominantly in the area of speech. The aim of the study was to explore the lived experiences of parental caregivers of children with a communication disorder in Johannesburg, South Africa.

## Research methods and design

A qualitative, interpretive phenomenological analysis (IPA) approach was used in the referred study to gain an in-depth, descriptive exploration of the participants’ experiences (Brocki & Wearden, 2006; Smith & Osborn, 2015). The participants were purposively selected from caregivers whose children attend a developmental preschool for children with communication disorders (Chapman & Smith, 2002; Patton, 2002). The preschool is a privately funded, nonprofit organisation in Johannesburg, South Africa. This urban site was chosen given that early intervention services may be more accessible in a city context, compared to a rural setting.

Studies that use IPA seek to recruit an analogous set of participants who hold a range of views on a particular subject, rather than a strictly representative sample (Flowers et al., 2006). Specific inclusion criteria included: (1) adult caregivers over 18 years, (2) the primary caregiver who is principally responsible for the majority care and upbringing of the child, (3) caregiver to a child between 3 years and 7 years (in line with the age of children attending the preschool), (4) the children attending the preschool, (5) the children presenting with a moderate communication disorder in hearing and/or speech and language development – this was diagnosed by a medical professional, (6) the children exhibiting impairments interfering with communication or are inconsistent with developmental age across communication situations, and (7) the communication disorder being the primary diagnosis. Please see Table 1 above for details of participants. Data were collected using a demographic questionnaire and individual, semi-structured interviews (Smith & Osborne, 2007). Interviews were conducted by the first author and were audio-recorded. These were held at the school or the University of the Witwatersrand’s Emthonjeni therapy centre and lasted between 45 min and 75 min each.

**TABLE 1:** Participants’ demographics.

Pseudonym	Age (years)	Relationship	Education	Work	Child’s age (years)	Disability
Anne	40	Married	Diploma	No	5†	Speech
Beck	32	Married	Matric	No	6	Hearing
Charlotte	37	Married	University	Full time	3	Speech
Diane	50	Married	University	Part time	7†	Speech
Elle	38	Married	University	No	7	Speech
Fran	24	Married	Matric	Part time	3	Speech

† , twins.

### Ethical considerations

Ethical approval for the study was granted by the Human Research Ethics Committee at the University of the Witwatersrand (reference no. MACC/18/002 IH). All participants provided written consent and have been given a pseudonym with all identifying information changed for anonymity and confidentiality purposes.

The sample consisted of six participants who are all women, white, middle class and the biological mother of their child with a communication disorder.

Analysis was undertaken by the first author using IPA. A systematic qualitative, case-by-case, inductive analysis of transcripts was conducted to detail the experiences and understandings of each individual case, before a broader, generalised understanding of the specific group of participants was sought (Smith & Osborn, 2007). The second author reviewed the analysis as a cross-checking exercise.

## Results and discussion

### Feeling out of control

The participants recounted when they first became aware of their children’s communication disorders, recalling this initial phase as being filled with various conflicts and challenges. They reported needing a variety of information regarding the relevant communication disorder. This need seemed to be related to their sense of guilt that they may not be doing enough to assist their children:

‘I couldn’t believe I missed it [*the communication disorder*]. Yeah, and I don’t work with children, but I should’ve known better’ (Charlotte, 37 years, Married, University).

The participants felt that if they did not act, they may not receive assistance or provide their children with the best solutions. This points to the lack of control that caregivers may experience in receiving a diagnosis of a communication disorder for their children. A participant recounted:

‘Nobody was telling me anything … Every step I took was because of my own research. Nobody ever even gave me a suggestion on what to do. Not one specialist.’ (Anne, 40 years, Married, Diploma)

Furthermore, this quote suggests a lack of support and guidance that the caregivers of children with communication disorders experience in relation to the healthcare system in South Africa.

Despite participants noticing the delays at an early stage, they experienced their concerns as being minimised, or even unheard by healthcare practitioners and educators. Van der Linde et al. (2016) express that the identification of communication disorders by healthcare workers in South Africa is often poor. In the current study, the professionals’ authority seemed to overrule the knowledge of caregivers, leaving them feeling out of control (Longmore, 2016). Caregivers often felt unheard and misunderstood by healthcare professionals, educators and family members during the period of early identification. Ignoring their maternal instinct led to delays in receiving a diagnosis and/or intervention for their children. Participants suggested that it is better to be overly concerned than ignore delays that may impact children at a later stage:

‘If you feel there is a problem you … stick to it, because people don’t listen, not even the doctors want to listen … stick to your guns and follow what your gut tells you … follow your instinct.’ (Beck, 32 years, Married, Matric)

‘[*W*]hat I have learnt as a mother which is very important is to just trust your gut. I think you can’t underestimate … like how important a mother … there are just things you know.’ (Fran, 24 years, Married, Matric)

The caregivers reflected on their frustration in engaging and coping with the unknown aspects of receiving a diagnosis of a communication disorder. Not knowing what the correct approach was or where to access help left them feeling anxious, helpless and hopeless:

‘I think the hardest thing for my husband through all of this [*receiving a diagnosis*] is he kept, he just wanted someone to say to him like, what does this mean, going forward, like he wanted answers …’ (Charlotte, 37 years, Married, University)

‘I think the thing that comes up as a parent is the unknown, there is a fear of, of you know, will my children speak? … because the world out there is not always easy and so it was a little bit, it was very anxiety provoking for me at times …’ (Diane, 50 years, Married, University)

Consequently, the participants’ mental health was impacted, leading to emotional breakdowns and suicidal thoughts:

‘[*A*]nd I said if this [*operation*] doesn’t work out I am committing suicide tonight … I couldn’t anymore.’ (Beck, 32 years, Married, Matric)

‘[*I*] had an emotional breakdown. I couldn’t, I couldn’t do this by myself.’ (Anne, 40 years, Married, Diploma)

### ‘I felt like I was getting nowhere’ – Systemic parental disempowerment

Participants shared their experiences of systemic disempowerment during the process of early identification and assistance seeking. Vergunst (2016) reiterates that services in South Africa are often formulated without taking into consideration the thoughts and emotions that shape the experience of disability. The participants conveyed difficulty in exercising their decision making, problem solving and interacting with others when accessing needed resources – both in the mainstream and specialist settings. This left them feeling unheard, rejected, alone and disempowered in their pursuit to assist their children. Beck reported that when she asked the doctor why her son is not talking, she received a dismissive response of:

‘[*A*]h no it’s a boy thing, boys take longer’ (Beck, 32, married, matric).

Saloojee (2008) also found that caregivers and their children with disabilities across South Africa are disempowered, passive recipients of health and therapy programmes; thus, Saloojee’s findings, while dated, remain relevant.

Professionals were experienced as unaware of the significant role that they can play in alleviating the feelings of parental frustration and powerlessness. Participants detailed how healthcare professionals insinuated paranoia in their anxieties, which left them questioning whether they were ‘crazy’, negatively impacting their confidence. This contributed to a sense of parental guilt:

‘[*N*]o one’s giving me answers, and everyone looks at me as if I am going crazy ….’ (Beck, 32 years, Married, Matric)

‘[*P*]rofessional people and medical people who will tell me that like I am crazy and … just wait it out …’ (Fran, 24 years, Married, Matric)

Although research emphasises the pros of early identification (WHO, 2012), participants remembered being repeatedly encouraged by healthcare professionals to give their children more time after they had identified delays. This appeared to exacerbate the caregivers’ frustration because they were feeling unheard and becoming increasingly concerned for their children. They experienced practitioners as withholding information. Additionally, caregivers felt judged for expressing their concerns:

‘No one’s listening to me.’ (Beck, 32 years, Married, Matric)

‘I went to a neurologist … she kept saying to me give them another six months …’ (Diane, 50 years, Married, University)

Despite there being an emphasis on the importance of early identification and intervention, caregivers felt that the healthcare system was ill-equipped to assist and support them in this process. Participants reported relying on educated guesses, at times requiring them to assert their own opinions, leaving them feeling untrusting of healthcare professionals:

‘[*M*]y choice, I say no because … I go to operate on my child [*opt for the operation*] for four hours and it’s going to come back not working.’ (Beck, 32 years, Married, Matric)

‘The problem is you [*audiologist*] don’t know what you actually saying.’ (Beck, 32 years, Married, Matric)

‘[*W*]hat really angered me is she couldn’t tell me what any of those other issues actually were that she wanted to rule out.’ (Charlotte, 37 years, Married, University)

Participants implored professionals to consider the disempowered and desperate position of caregivers and contemplate the implications of their professional actions and suggestions:

‘[*A*] message to the people involved … just think, do you really have to put us through what you putting us through? Is it really going to tell you anything that you don’t already know? Because if it isn’t, like please don’t put us through that just so that you can feel you ticked off a box …’ (Charlotte, 37 years, Married, University)

### Need for support

Participants often experienced difficulty in finding support for, and coping with, the emotional burden of caring for a child with a communication disorder. Participants felt that this was systemically based in the South African context where formal support services were perceived to be lacking:

‘[*I*]n South Africa … I feel like there is no support system … there is no support group’ (Anne, 40 years, Married, Diploma).

Participants reported having to access private support.

Furthermore, participants struggled to rely on available resources in fear of becoming a financial burden to their families. They tried to uphold an appearance of coping without accessing support services in order to mitigate further ramifications to the already overburdened family system because of the presence of disability:

‘[*T*]here’s no support groups for them [*moms*]. If they have to pay for going to see somebody, then they going to have to take away from their family financial situation and we all know that moms put themselves last.’ (Anne, 40 years, Married, Diploma)

Despite formal support structures appearing limited and poorly accessed, the caregivers placed emphasis on the importance of having such structures. They regarded the platform of shared experience with other caregivers of children with communication disorders as an imperative form of informal support and understanding. They experienced these communities as nonjudgemental guiding spaces:

‘I have made a mommy friend here [*pre-school*] … and the two of us when we talk it’s so amazing … because usually we feel alone … and it doesn’t matter if your child has hearing aids, or cochleas, or nothing, we have all got the same stories that [*laughs*] our children don’t talk.’ (Beck, 32 years, Married, Matric)

‘[*W*]ithin my mom friends just being open about it, it’s allowed them to support me on a huge level which has been incredibly helpful just having that emotional support.’ (Charlotte, 37 years, Married, University)

‘And then also your friends you make at the school. You become a family … It’s been an enormous thing.’ (Diane, 50 years, Married, University)

### Barriers to accessing services

The participants reported experiencing multiple barriers in accessing services, providing some insight into the systemic context within the South African setting.

#### Financial barriers

Participants articulated their vulnerability to the cost of having a child with a communication disorder. Costs include therapies, specialised schooling, healthcare services and medical aid:

‘[*F*]inancial restraints … the school for instance. It’s the same cost as a private school for your child. I pay less for my child in university than what I paid for my kids in pre-primary.’ (Anne, 40 years, Married, Diploma)

‘It put pressure on you financially, because suddenly you have got to … do stuff that you, you wouldn’t have, you weren’t expecting.’ (Diane, 50 years, Married, University)

‘[*T*]he main thing that’s affected is your financial status because what you need to pay per month for therapies and the insurance, medical aid doesn’t actually cover anything.’ (Elle, 38 years, Married, University)

The participants seemed to experience financial stresses stemming from the lack of comprehensive social protection systems in South Africa, whereby families felt unsupported by government-based services. They felt as if the available services were often inadequate for children with communication disorders; therefore, they felt compelled to access additional private assistance to provide adequate intervention for their children:

‘[*F*]rustrating … a lot of the therapies get covered through your [*medical aid savings*]. But once that’s done you … pay the gap … I think the country doesn’t assist or medical aid … doesn’t assist these kids with special needs …’ (Elle, 38 years, Married, University)

‘[*O*]ther than a country not being able to cater for kids with speech disabilities. Not having enough … intervention, not having enough, financial intervention … very tricky … because it’s not a normal disability.’ (Elle, 38 years, Married, University)

#### Limited resources

Participants relayed experiencing limitations in accessing amenities to assist their children because of scarcity of resources, the vast geographical location of resources, limited specialists and long waiting lists. They articulated that it required additional time, effort and cost to actively seek out and access resources because of minimal and often inaccessible placement of schools and healthcare professionals across various geographical areas in Johannesburg. Research has found that there is limited access to appropriate interventions in the public health and education sectors in South Africa despite policy reformation to increase services (Malcolm Smith et al., 2013). The current study’s participants also experienced that the situation has not changed since this early study. Saxena et al. (2007) also reported that South Africa has severely limited access to knowledge and activities directed towards childhood disabilities, resulting in a scarcity of available resources, inequities in the distribution of resources, and inefficiencies of the use of resources to meaningfully assist children with disabilities and their carers. Furthermore, 67% of registered speech and language practitioners in South Africa are in private practice (Kathard & Pillay, 2013). Additionally, participants reported limitations in access because of a lack of structural facilities and intervention personnel:

‘[*T*]hing that I find challenging is you don’t find a lot of schools or places that accommodate children of cochleae or hearing aids or kids with hearing problems in general … everything else is …quite far.’ (Beck, 32 years, Married, Matric)

‘I think I booked in July or June, and we saw the doctor in September … Because there is so few of them, it’s a very long wait.’ (Charlotte, 37 years, Married, University)

‘So even though he is very young I have put his name down [*at a Remedial School*] … because the waiting list is … It’s almost impossible to get in.’ (Charlotte, 37 years, Married, University)

The participants were all white, middle class with access to both public and private spaces; however, they relayed that despite having financial privileges to access services, they still experienced limitations in resources within the South African setting. They were able to acknowledge their privilege and questioned what others without such advantages might do:

‘But what do you do if you can’t afford it [*schools*], what do you do if you don’t know about it? … what would’ve happened to a child … if we relied on Government systems, like what would actually have happened to him?’ (Charlotte, 37 years, Married, University)

‘I don’t know how people do manage, but I think being in [*a big city*] in terms of resources has been I think a big plus … I don’t know if there are these kind of schools in other parts of the country.’ (Beck, 32 years, Married, Matric)

Horton and Shakespeare (2019) report that specialised schools are advantageous because they meet disabled children’s needs and allow children to meet other people with disabilities and to find motivation from their peers and teachers. However, the participants’ preference for specialised schooling points to the failure of inclusive education policies to address barriers to learning in the South African education system as a strategy that can contribute to furthering destigmatisation and increasing inclusivity in a democratic society. Participants articulated feeling rejected by mainstream schooling services because of misinformed perceptions, social exclusion and poor efforts to assist children with disabilities within their capabilities:

‘And you also don’t realise what happens within the school system. You pay the school thinking that you know the kids are being looked after and protected and they not, they generally not, the school systems are not run properly here [*in SA*]. And there is no integration … there is supposed to be what, special needs integration, there is none of that.’ (Elle, 38 years, Married, University)

Literature speaks to the risks of facing social exclusion, stigmatisation and a lack of understanding especially towards a child facing communication disorders (Castellani et al., 2021). Swart et al. (2002) agree that the establishment of inclusive schooling requires more than the implementation of new policies, and a notable shift must be made in providing ongoing support and in-service training to teachers. In addition, the caregivers of the current study identified a need for a systemic change at a broader level that addresses the social stigma of children with disabilities in order to promote inclusion. However, they feared utilising their own children in this process as they did not want their children being left out, hurt by questions, dealing with frustration, trying to fit in, failing and encountering discouragement. The parental caregivers reflected on this double bind, acknowledging how overprotecting and sheltering their children from the mainstream setting leads to disadvantage, exclusion and stigmatisation that further perpetuate the common narrative on disability.

#### Limited information on services

Participants felt that seeking services was difficult because of a secrecy around accessing these. They struggled to find information on available resources and intervention services, placing them under additional stress. This left them feeling alienated and powerless because of the perceived lack of support and guidance from stakeholders, particularly within the public systems. Caregivers felt further disempowered because this limited their choices:

‘I had to find out about the schools through different means …’ (Anne, 40 years, Married, Diploma)

‘I know the school from, from when I studied and I did a prac[*tical*] there, like I don’t think a lot of people know about it.’ (Charlotte, 37 years, Married, University)

‘[*T*]here is no organisation really that help out with these school things, the Government is sort of, I don’t know. Somewhere else.’ (Anne, 40 years, Married, Diploma)

The participants’ experiences reiterated the findings that childhood communication disorders remain relatively unknown and underresearched in comparison to other disabilities, with a focus on intervention, while the areas of prevention and health promotion have remained underresearched (Maulik & Darmstadt, 2007; Simms & Jin, 2015). The focus on intervention in favour of prevention and health promotion places additional burden on the healthcare system, as well as on caregivers.

#### Overreliance on private sector

The segmentation of the healthcare system was apparent, with most participants perceiving public healthcare interventions to be inadequate, preferring to access private healthcare, despite the additional cost thereof. Caregivers believe that the poorly functioning public healthcare sector caters primarily to the poor, while private healthcare institutions offer better quality interventions that satisfy the more affluent. Therefore, paying for an intervention is believed to elicit better services:

‘If I want something and then I had to pay for it … I obviously have to pay the private sector for it because it’s the only people that’s going to help me.’ (Anne, 40 years, Married, Diploma)

Participants further highlighted a perceived reduction in the role of the state in the provision of healthcare services, with the transfer of formerly provided state services to the private sector. They experienced the overreliance on the private sector as undermining investments in improvements in primary state healthcare and therefore eroding the government’s commitment to the right to health. Participants are left feeling unsupported by the South African Government and primary healthcare system:

‘You’re on your own … I don’t think there is much help from the Government sector. Yeah, there is no help from the Government sector’ (Elle, 38 years, Married, University).

Studies found similar concerns regarding access to services for children with communication disorders in South Africa because of them having low priority in healthcare systems; their interventions often straddle the professions of education and health, and they are not always fully understood by each sector; resources are limited, and the professions are small, new or unknown (Nippold, 2010; Pascoe & Norman, 2011; Van der Linde et al., 2016).

### Caregivers left speechless

The caregivers described having a child with a communication disorder as emotionally overwhelming. Although feeling intense emotions can be considered an expected aspect of parenting young children, the caregivers expressed that their emotions were often intensified in reaction to their children’s communication disorders and accompanying developmental delays. These emotions were often described through a rainbow of ambiguity:

‘[*I*]t’s been a mix of anxiety, and it’s lot of emotions, but then also a lot of joy …’ (Diane, 50 years, Married, University).

Although the experience of raising a child with a communication disorder is accompanied by both positive and negative feelings, many of the caregivers were overwhelmed by negative emotional experiences, particularly feelings of worry. The extent of their concern often left them feeling as if words were inadequate to describe the immense fear that they carry for their children:

‘I don’t think there is anything positive about apraxia.’ (Elle, 38 years, Married, University)

‘[*W*]ords sometimes are a little bit inadequate for the … the internal stuff around what happens to one … it’s probably been one of the most, up until now, one of the most worrying things that’s happened …’ (Diane, 50 years, Married, University)

‘You worry. You worry about their future.’ (Elle, 38 years, Married, University)

Participants also shared how the burden of their worry and frustration impacted on their relationships with their children because they were unable to resolve their children’s discomfort, confronting caregivers with a sense of impotence and lack of control:

‘I felt useless, absolutely useless … Until people actually finally got me an answer then how can I go back and fix it.’ (Beck, 32 years, Married, Matric)

Much of the responsibility of caring for a child with a communication disorder appeared to stem from the need for additional therapies. Caregivers felt great responsibility to not only get their children to therapy but also to sit in on these sessions and be involved in the intervention process. Many participants expressed the difficulties of being a child’s therapist in addition to being their mother, resulting in stressful emotional experiences that left caregivers feeling blamed for hampering their children’s therapeutic processes:

‘[*T*]he worst part … to sit in every session, OT, Speech, and … I was actually saying to his Speech Therapist at school, it was the most emotionally draining, I used to dread it, because first of all you watching him struggle … or they have a meltdown or they come to you or they don’t do what’s being asked of them … that for me was too much pressure. I hated it …’ (Charlotte, 37 years Married, University)

### A misunderstood disability

Participants articulated that communication disorders tend to be hidden disabilities, as they do not necessarily manifest as physically apparent, easily identifiable in the public eye. They expressed that many people had never heard of communication disorders, including trained healthcare professionals and educators:

‘Apraxia … most people, even today, it’s one of those well what is that … General Practitioners don’t [*know*], and they kind of look at you’ (Diane, 50 years, Married, University).

The caregivers conveyed how their children appeared able-bodied to others; however, their communication frustrations often presented through behaviour difficulties that drew attention to them in public and were interpreted as misbehaviour. The participants are left feeling judged and angered by others, indicating a misunderstanding towards people with invisible disabilities:

‘[*P*]eople aren’t aware of it, when they have mainstream kids … There is no empathy … unless your kids have a disability you not aware of, I mean before [*my child*] I didn’t know about apraxia. But I mean I wouldn’t be so harsh as to say keep your child’s volume down ….’ (Elle, 38 years, Married, University)

Caregivers felt that their children are pressured into upholding social expectations because of their able-bodied appearance. They shared how much of the difficulties they faced in social settings stemmed from this lack of awareness because of the often-hidden nature of communication disorders:

‘It is a frustrating disability … but on the other hand I am grateful that it’s not something immediate that you can judge a person by.’ (Fran, 24 years, Married, Matric)

‘[*W*]e lucky that it’s a, it’s a speech disability, it’s not, God forbid, a physical disability … It’s a very strange disability because your kids are one hundred per cent fine.’ (Elle, 38 years, Married, University)

The caregivers reflected on the largely negative and misinformed social perceptions and narratives of disability in South Africa. These narratives appeared to be rooted in the medical model of disability and largely hidden from society. This is believed to stem from the public’s misconceptions, prejudices and stereotyping towards persons with disabilities (Swartz & Watermeyer, 2006). This is in line with the findings of the African Child Policy Forum (ACPF) (2011): in South Africa, disabled children and their families are often socially excluded and denied opportunities for participation, resulting in multiple deprivations and invisibility in the policy agenda. Furthermore, literature shows that there continues to exist prejudice and stigmatisation of disability because disability in South Africa continues to be situated in the individualistic medical model (Gathiram, 2008; Swartz & Watermeyer, 2006; WHO, 2011).

Moreover, the common social narrative suggests that people seem to assume a lowered expectation of achievement and participation in children with disabilities. Disabling social perceptions lead to disabling barriers that characterise these caregivers’ and their children’s social encounters:

‘I don’t want people to treat him like he is stupid because he is not stupid, and he understands everything … I don’t want people to not talk to him and not engage with him.’ (Charlotte, 37 years, Married, University)

The lowered social expectation of children with disabilities resulting in social exclusion appeared to be further impacted by parental overprotection. Participants articulated that they did not want their children ignored or emotionally hurt:

‘I didn’t want to send them to pre-school, because they weren’t communicating properly, and I was scared in such a big situation they don’t just get lost.’ (Anne, 40 years, Married, Diploma)

‘The more vulnerable they are the more you got to protect them … I’ve got to constantly watch out that they ok … people have bad day, teachers have bad days with ‘normal’ children. Can you imagine if they have it with children that can’t fend for themselves or can’t tell me that you did this to them?’ (Anne, 40 years, Married, Diploma)

‘I think it’s made me more protective. Especially … about leaving him with other people, that fear that they wouldn’t understand him …’ (Charlotte, 37 years, Married, University)

Participants reflected on how they attempt to shelter their children by intervening in order to prevent judgement. They take over tasks for their children, rather than assisting them in navigating the task for themselves, thereby yielding them less independence:

‘I would rather get ahead of the curve and tell people what’s wrong with [*child*] than let them jump to their own conclusions … you don’t have to not tell people or like hide it away … I feel like we are his voice, and we are his advocate.’ (Charlotte, 37 years, Married, University)

### Relinquishing control: *‘I needed to be a mommy’*

The participants reflected on the process of meaning-making and acceptance they went through in receiving a diagnosis of a communication disorder for their children. A diagnosis resulted in the rise of many difficult emotions associated with grief and loss, which is common in the literature (Harvey, 2015). Initially, caregivers felt an overwhelming responsibility to ‘fix’ their children. Through a process of acceptance, they were better able to manage their emotions and let go of this sense of responsibility and fear of failure. The caregivers were able to identify and make changes to their personal narratives that were inhibiting their acceptance and enjoyment of their children. This allowed them to reformulate their own identities and draw on positive familial resources.

The caregivers’ overinvestment in their children’s therapeutic process often left their children feeling overwhelmed, overstimulated and rejected. The need to ‘fix’ their children was further driven by social perceptions on caregivers’ involvement in early intervention. However, the focus on ‘fixing’ their children left little space for the caregivers and their children to have fun and be themselves:

‘I just wanted to come home from work … I am tired, he is tired, I don’t want to do therapy with him … I don’t want to do it with him, and I don’t want him to feel like everything with me is work. I just want to do normal stuff.’ (Charlotte, 37 years, Married, University)

‘[*Y*]ou just want to come home, you want to let the kids be kids and they have to, now … to another Speech Therapist.’ (Elle, 38 years, Married, University)

Participants recognised that much of their emotional distress stemmed from their denial and reluctance to accept their children’s disabilities. Accepting their children and their difficulties required them to relinquish the conventional belief of families coping with raising a disabled child as situated in crisis, stress and pathology. Participants expressed that they had to let go of trying to ‘fix’ their children by accepting that which they had so often denied:

‘I just woke up one morning and said I can’t emotionally deal with this anymore. Whatever will be, will be and I am going to have to swallow this pill and I am going to have to be a mommy to them. I am going to have to be a person that they can feel that they can come and hug and kiss instead of being this rigid therapist.’ (Anne, 40 years, Married, Diploma)

In adapting to, and accepting, their children, the caregivers were better able to let go of their need to control. They expressed that they were better equipped to emotionally connect with their children and others, which stemmed from a lack of judgement and expectation. This resulted in an emotional adjustment towards an increased perception of acceptance:

‘[*I*]t’s hard when you can’t control something …’ (Diane, 50 years, Married, University)

I think it teaches you so much about yourself and expectation and control, and it forces you to live in the moment and really appreciate …’ (Diane, 50 years, Married, University)

Through accepting their children, they were able to relinquish their need to control and allow them to be themselves while supporting their children. The caregivers were better able to identify strengths and capabilities within their children that allowed for acceptance, a stepping back from responsibility and an enjoyment of their children through a process of autonomy acquisition:

‘[*I*]t’s been … a process that we have done a lot together … I had to let go … so it was something that I had to as a mother … sort of let go’ (Fran, 24 years, Married, Matric).

### Limitations

Smith et al. (2009) advise researchers to recruit a fairly homogeneous sample of participants in IPA studies, which is true of the parental caregivers in this study, as they share similar traits in terms of their age, gender and background. However, this may be considered a limitation of the study because the sample was predominated by white females that stemmed from a middle-class background.

## Conclusion

The article presents a rich picture of caregivers’ experiences of raising a child with a communication disorder in the South African city context of Johannesburg. Despite this experience having both positive and negative emotional aspects, many of the caregivers were overwhelmed by, and focussed on, negative emotional experiences. This was particularly apparent in the early stages of their journey, which was marred by worry and fear of the future. Parental caregivers appeared to focus on attempting to fix their children through accessing intervention services that have often been essentially oppressive in nature and epidemiologically focussed (Longmore, 2016).

In accessing services, the caregivers narrated their experiences of multiple barriers to access, including an overreliance on the private sector, financial barriers, time constraints, limited resources and poor information on available resources. Caregivers and their children with disabilities in South Africa continue to be disempowered, passive recipients of health and therapy programmes that focus on epidemiology and intervention instead of being person-centred, multidisciplinary and supportive of healthcare promotion (Saloojee, 2008). Barriers to care may marginalise and seclude caregivers. Over time, the caregivers were able to empower themselves through building their own knowledge and reformulating their narratives by challenging assumptions. They were better able to identify strengths, skills and capabilities within their children and themselves that allowed for adjustment and coping.
